# Ebselen prevents cigarette smoke-induced cognitive dysfunction in mice by preserving hippocampal synaptophysin expression

**DOI:** 10.1186/s12974-022-02432-y

**Published:** 2022-03-29

**Authors:** Simone N. De Luca, Kurt Brassington, Stanley M. H. Chan, Aleksandar Dobric, Kevin Mou, Huei Jiunn Seow, Ross Vlahos

**Affiliations:** School of Health and Biomedical Sciences, RMIT University, PO Box 71, Bundoora, VIC 3083 Australia

**Keywords:** Cigarette smoking, Cessation, Neuroinflammation, Cognition, Antioxidants, Synaptogenesis

## Abstract

**Background:**

Cigarette smoking (CS) is the leading cause of chronic obstructive pulmonary disease (COPD). The “spill-over” of pulmonary inflammation into the systemic circulation may damage the brain, leading to cognitive dysfunction. Cessation of CS can improve pulmonary and neurocognitive outcomes, however, its benefit on the neuroinflammatory profile remains uncertain. Here, we investigate how CS exposure impairs neurocognition and whether this can be reversed with CS cessation or an antioxidant treatment.

**Methods:**

Male BALB/c mice were exposed to CS (9 cigarettes/day for 8 weeks) followed by 4 weeks of CS cessation. Another cohort of CS-exposed mice were co-administrated with a glutathione peroxidase mimetic, ebselen (10 mg/kg) or vehicle (5% CM-cellulose). We assessed pulmonary inflammation, spatial and working memory, and the hippocampal microglial, oxidative and synaptic profiles.

**Results:**

CS exposure increased lung inflammation which was reduced following CS cessation. CS caused spatial and working memory impairments which were attributed to hippocampal microglial activation and suppression of synaptophysin. CS cessation did not improve memory deficits or alter microglial activation. Ebselen completely prevented the CS-induced working and spatial memory impairments, which was associated with restored synaptophysin expression without altering microglial activation.

**Conclusion:**

We were able to model the CS-induced memory impairment and microglial activation seen in human COPD. The preventative effects of ebselen on memory impairment is likely to be dependent on a preserved synaptogenic profile. Cessation alone also appears to be insufficient in correcting the memory impairment, suggesting the importance of incorporating antioxidant therapy to help maximising the benefit of cessation.

## Introduction

Chronic obstructive pulmonary disease (COPD) is a major incurable global health burden affecting 210 million people worldwide and is the 3rd leading cause of death with cigarette smoking (CS) being the major cause in industrialised countries [1]. Noxious particles in CS damage the lung epithelium, driving the recruitment of macrophages, neutrophils, and lymphocytes as well as the release of pro-inflammatory mediators [2–4] and reactive oxygen species (ROS) [5, 6] into the lungs, that drives persisting pulmonary inflammation. Pharmacotherapies targeting oxidative stress, such as the glutathione peroxidase (GPx) mimetic, ebselen, can ameliorate lung inflammation in murine models of COPD [7–10]. It is postulated that injuries of the lung epithelium permits the “spill-over” of pro-inflammatory and oxidative stress mediators into the systemic circulation, leading to secondary medical conditions elsewhere, also known as comorbidities [11].

COPD-induced neurocognitive dysfunction is an emerging area of research which includes alterations in memory, executive functioning and attention [12–14], with up to 61% of people with COPD suffering from neurocognitive dysfunction [12, 15, 16]. Moreover, neurocognitive dysfunction is associated with much of the disease burden, healthcare utilisation and costs, as people with neurocognitive dysfunction often lack adherence to therapeutic interventions and medication, which in turn worsen their COPD morbidity, lead to increased hospitalisations and risk of mortality [12, 17]. Currently, CS cessation is the most effective strategy for improving respiratory symptoms and function in people with COPD [18–21] and may improve cognitive function as soon as 2 years post-quitting [22–24].

Although the “spill-over” hypothesis may provide an overarching theme for the onset of comorbidities in COPD, the molecular pathway underlying COPD-related neurocognitive dysfunction remain unknown. One possibility is the potential link between CS-induced neurocognitive dysfunction and microglial-mediated neuroinflammation. Microglia are resident immune cells of the brain that modulate and support neurogenesis, synaptogenesis and cognition in healthy individuals [25]. In response to perturbations, microglia undergo morphological transition from a surveillant ramified state to an activated state adapting the ameboid morphology in response to injury, phagocytosing cellular debris [26, 27]. However, chronic activation of microglia can have deleterious effects, promoting neuronal and axonal loss, which would eventuate in neurocognitive dysfunction [28, 29]. Furthermore, studies have shown that microglia are not only sensitive to oxidative stress, but activated microglia are also capable of producing ROS themselves, thereby perpetuating the oxidative burden [30] and neurocognitive dysfunction seen in COPD.

Microglia-driven neuroinflammation is a major mechanism for cognitive dysfunction, however, whether this type of neuroinflammation is reversible by CS cessation is unknown. Therefore, we investigated the effect of CS cessation on cognition, neuroinflammatory and oxidative stress profiles in the hippocampus of mice to model that of ex-smokers. We also evaluated the effect of co-administration of the antioxidant, ebselen, in memory performance and the neuroinflammatory profile post-CS exposure.

## Materials and methods

### Animals

All animal care and experimental procedures were in compliance with the ARRIVE Guidelines [31], Australian Code of Practice for the Care of Experimental Animals and RMIT University Animal Ethics approval (AEC1533). Male BALB/c mice obtained from the Animal Resource Centre (WA, AUS) arrived at 7 weeks of age. The mice were kept under standard laboratory housing conditions, with a 12-h light/dark cycle, an ambient temperature of 22 °C and ad libitum access to water and standard mice chow.

### Cigarette smoke exposure and ebselen treatment

Mice were randomly assigned into different experimental groups (*n* = 14/group) and body weight was recorded every second day. Mice underwent whole body exposure to the smoke of 9 filtered cigarettes per day (Winfield Red, 16 mg or less of tar, 1.2 mg or less of nicotine),  or mice were exposed to room air (sham) for 5 consecutive days a week for 8 weeks as previously published [32, 33]. Cohort 1 consisted of four groups exposed to CS and one group exposed to room air. Cohort 2 were exposed to CS or room air for 8 weeks with co-administration (oral gavage) of either ebselen (10 mg/kg) or 5% CM-cellulose (vehicle). After 8 weeks of exposure, mice were culled by an overdose of anaesthetic (Lethabarb, 240 mg/kg; Virbac Pty. Ltd.) or culled after a cessation period of 3, 10 and 33 days.

### Bronchoalveolar lavage and lung collection

At the end of the study, the lungs were lavaged in situ using phosphate buffered saline (PBS) as previously published [7, 32, 34]. Bronchoalveolar lavage fluid (BALF) cytospin slides were prepared and analysed; identifying macrophages, neutrophils and lymphocytes using standard morphological criteria. The lungs were then perfused free of blood with PBS, rapidly excised en bloc, blotted and the large left lobe snap frozen for quantitative real-time-PCR (qRT-PCR) analysis.

### Brain dissections

Brains were sagittally hemisected and the right hemisphere was excised and the hippocampi was snap-frozen for gene or protein expression (*n* = 6/group). The left hemisphere was immersed in 4% paraformaldehyde in PBS for 24 h before cryoprotecting in 20% sucrose (*n* = 6–8/group) for histological analyses. Brains were sectioned into 30 µm coronal sections in a one-in-five series using a cryostat (Leica Biosystems, VIC, AUS).

### Gene expression

Total RNA was extracted using RNeasy kits (QIAGEN, CA, USA), then reverse transcribed using a High-Capacity RNA-to-cDNA kit (Life Technologies, CA, USA) prior to qRT-PCR analysis (QuantStudio 7, Applied Biosystems, VIC, AUS). All reactions were performed in triplicate, and the data obtained were normalised to *Gapdh* as an endogenous control before analysis using the ∆∆CT method (Table 1).

**Table 1:** TaqMan probe details (Life Technologies) used for qRT-PCR

Target gene	NCBI reference sequence	TaqMan assay ID	Amplicon size (bp)
*Tnf*	NM_013693.3	Mm00443258_m1	81
*Il6*	NM_031168.1	mm00446190_m1	78
*Il1b*	NM_008361.3	Mm00434228_m1	90
*Ccl2*	NM_011333.3	Mm00441242_m1	74
*Cxcl1*	NM_008176.3	Mm04207460_m1	111
*Cxcl2*	NM_009140.2	Mm00436450_m1	67
*Mmp9*	NM_013599.3	Mm00442991_m1	76
*Mmp12*	NM_008605.3	Mm00500554_m1	78
*Tgfb*	NM_011577.1	Mm01178820_m1	59
*Csf1*	NM_007778.4	Mm00432686_m1	70
*Gpx1*	NM_008160.6	Mm00656767_g1	134
*Nox1*	NM_172203.2	Mm00549170_m1	84
*Cybb (Nox2)*	NM_007807.5	Mm01287743_m1	63
*Nox4*	NM_015760.5	Mm00479246_m1	72
*Nos1(nNos)*	NM_010927.3	Mm00440502_m1	66
*Nos2 (iNos)*	NM_010927.3	Mm00440502_m1	66
*Nos3 (eNos)*	NM_008713.4	Mm00435217_m1	71

### Immunohistochemistry

Coronal sections were blocked for 2 h at room temperature with 3% BSA, 10% NHS, 0.3% Triton X-100 in PBS and incubated in primary antibody (anti-Ionised calcium binding adaptor molecule-1 [Iba-1]: 1:1000, rabbit, Wako Chemicals, VA, USA; RRID: AB_2314666; anti-Synaptophysin: 1:2000, mouse, Sigma-Aldrich, MO, USA; RRID: AB_477523) overnight at 4 °C. Sections were washed in 1 × PBS-T then incubated with the fluorescent secondary antibody (1:400, anti-rabbit: Life Technologies, CA, USA; RRID: AB_221544; anti-mouse: Life Technologies, RRID: 2,536,161) for 2 h, mounted and coverslipped with Fluroshield DAPI mounting medium (Sigma-Aldrich). Hippocampal photomicrographs were taken on an upright fluorescent Olympus BX53 microscope (Olympus Corp., Tokyo, Japan). Three sections 60 µm apart between 1.46 and 2.54 mm relative to the bregma were analysed [35]. We assessed Iba-1 images for numbers and area per cell density [25]. For synaptophysin, immunofluorescent intensity was determined via CELLSENS imaging software (Olympus Corp). Detection thresholds were set to minimise background fluorescence and allow selection of an area of interest.

### Western blot

The right hippocampi from a separate cohort and BALF (*n* = 6/group), was homogenised (20 µg of total protein or 20 µL of BALF) and subjected to either SDS/PAGE using a 10% acrylamide gel or an OxyBlot™ Protein Oxidation Detection Kit (Millipore, MA, USA) [8]. Membranes were incubated overnight (4 °C) with primary antibody (anti-Malondialdehyde [MDA]: 1:1000, mouse, Thermo Scientific; RRID: AB_2735263; anti-GAPDH: 1:3000, rabbit, CST, QLD, AUS, RRID: AB_10622025; anti-DNP: 1:300, Millipore) followed by a 2 h incubation with HRP-conjugated secondary antibody (1:1000; CST; RRID: AB_330924). Membranes were then exposed to enhanced chemiluminescence Western Lighting Ultra Solution reagents (Perkin Elmer, MA, USA) and visualised using ChemiDoc (Bio-Rad Laboratories). Relative protein expression was normalised to the band intensity of GAPDH (MDA) or Ponceau S (Sigma-Aldrich) staining.

### Neurocognitive assessment

#### Spontaneous alternation in Y maze (sY-maze)

Animals were placed in a Y-maze with spatial cues external to the maze to enable spatial orientation and were freely allowed to explore for 5 min. An arm entry was recorded when the mouse had moved all four paws into an arm. The number of arm entries and the number of sequential entries into all three arms were recorded. The percentage of spontaneous alternation was calculated by dividing the number of alternations by the number of possible alternations.

#### Novel object recognition (NOR)

Mice were habituated to the arena for two sessions prior to testing (*n* = 8–10/ group). Mice were allowed to explore two identical objects placed equally distanced apart (8 min). Following a 1 h inter-trial interval, one object was replaced with a novel object (8 min). The preference index was calculated as time spent interacting with the novel object / overall exploration time of the two objects [36, 37]. Total distance was scored using Ethovision (Noldus Information Technology, Wageningen, NL).

### Data analysis

We analysed the data using a one-way analysis of variance (ANOVA) for cohort 1 and a two-way ANOVA for cohort 2. Where significant differences were found, we performed Tukey’s post hoc analysis. Body weights were analysed using repeated measures (RM) ANOVA with CS as the between factor and day as RM. Pearson’s correlation coefficient and regression analysis were used to evaluate the association between protein carbonylation and BALF total cell count, as well as preference index and total locomotor activity in the NOR and sY-maze test. Data are presented as the mean + SEM. Statistical significance was assumed where *p* ≤ 0.05.

## Results

### Smoking cessation restores body weight and reduces BALF cellularity

**Fig. 1:**
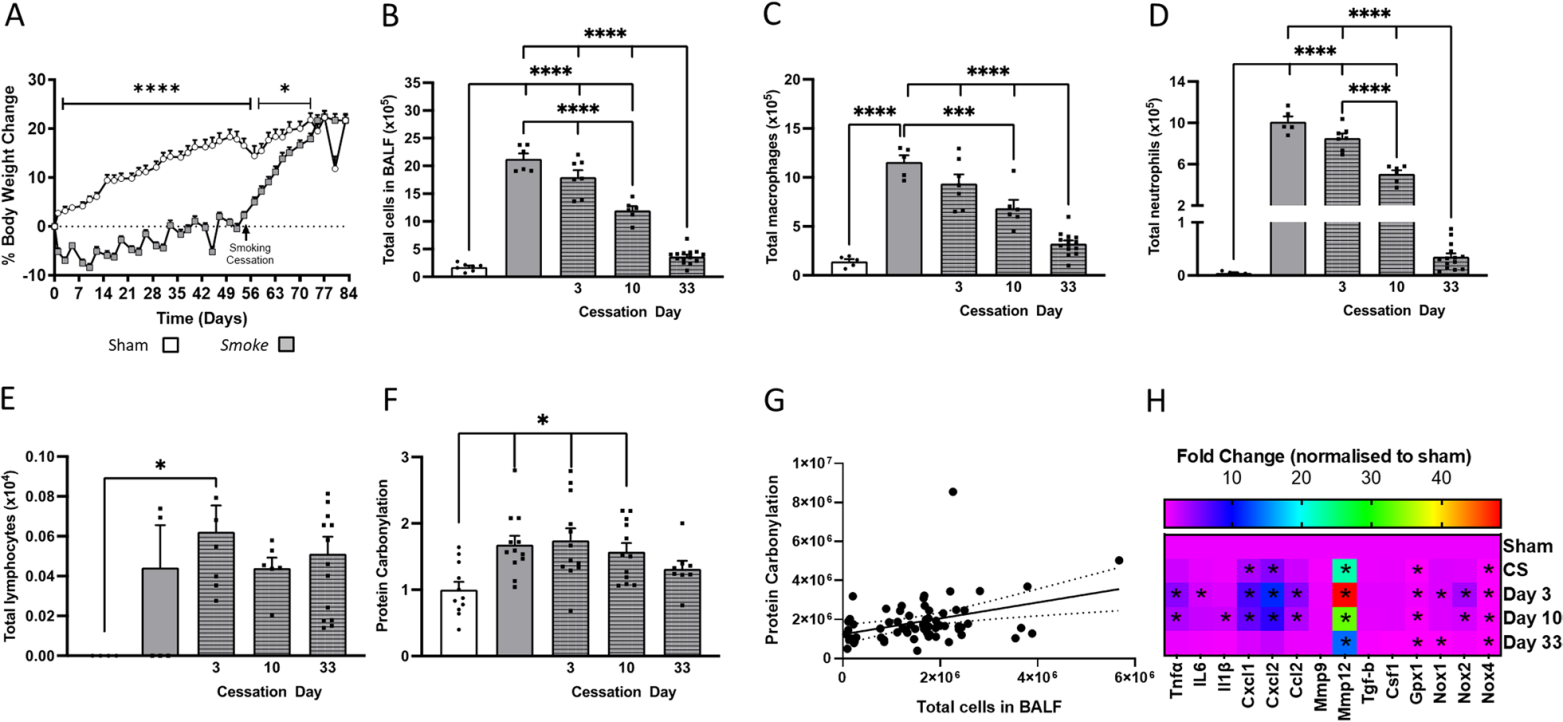
Cigarette smoke (CS)-induced weight loss and bronchoalveolar lavage fluid (BALF) cellularity was reversed 33 days after CS cessation. Mice were exposed to CS or room air (sham) for 8 weeks. CS exposure was then stopped, and mice were housed for an additional 3, 10 or 33 days. **A** Body weight percentage change (exposure by day interaction: *F*_(37,888)_ = 54.87, *p* < 0.0001; *n* = 14 per group). **B** Total number of cells (*F*_(4,35)_ = 146.1, *p* < 0.0001); **C** macrophages (*F*_(4,32)_ = 41.88, *p* < 0.0001); **D** neutrophils (*F*_(4,35)_ = 272.5, *p* < 0.0001); **E** lymphocytes in BALF (*n* = 6–10 per group). **F** BALF protein carbonylation (*F*_(4,50)_ = 4.553, *p* <  = 0.003) and **G** BALF protein carbonylation correlation (*r*_(62)_ = 0.1350, *p* = 0.0028; *n* = 6 per group). **H** Gene expression of cytokines and chemokines in the lung (tumour necrosis factor [*Tnf*]: *F*_(4,23)_ = 24.11, *p* < 0.0001; interleukin 6 [*Il6*]: *F*_(4,25)_ = 4.03, *p* = 0.011; *Il1b*: *F*_(4,25)_ = 5.83, *p* = 0.002; C-X-C motif chemokine ligand [*Cxcl*]-1: *F*_(4,23)_ = 7.90, *p* = 0.0004; *Cxcl2*: *F*_(4,21)_ = 61.69, *p* < 0.0001; C–C motif chemokine ligand 2 [*Ccl2*]: *F*_(4,23)_ = 24.11, *p* < 0.0001; Matrix Metalloproteinase [*Mmp*]12: *F*_(4,23)_ = 24.57, *p* < 0.0001; glutathione peroxidase [*Gpx*]1: *F*_(4,23)_ = 4.929, *p* = 0.0051; NADPH oxidase [*Nox*]1: F_(4,24)_ = 9.292, *p* < 0.0001; *Nox2*: *F*_(4,23)_ = 25.42, *p* < 0.0001; *Nox4*: (*F*_(4,25)_ = 6.360, *p* = 0.0011). Data are expressed as mean + SEM. * *p* < 0.05, *** *p* < 0.001, **** *p* < 0.0001

Similar to previously reported, CS exposure caused a suppression of weight gain when compared to sham mice [7, 32, 33]. Upon cessation, the body weight of the mice rapidly re-bounced and were no different to that of sham-treated at 18 days post-cessation (Fig. 1A). CS exposure caused an increase in BALF total cells (Fig. 1B), which was attributed to increased macrophage (Fig. 1C) and neutrophil recruitment (Fig. 1D) but not lymphocytes (Fig. 1E). Upon 33 days of cessation, BALF cellularity returned back to sham levels and this positively correlated with reduced BALF protein carbonylation (Fig. 1F, G).

### Cessation gradually alters the pulmonary inflammatory profile.

CS exposure significantly increased *Tnf*α expression in the lungs when compared to sham mice and this elevated expression was maintained immediately following cessation at both 3- and 10-day time points (Fig. 1H). CS prompted an increase in chemokines C-X-C Motif Chemokine Ligand (*Cxcl*)-1, *Cxcl2* and Chemokine ligand 2 (*Ccl2*) expression, however, this subsided back to sham levels following cessation. CS-exposure significantly increased matrix metalloproteinase (*Mmp*)12 expression when compared to sham mice despite a 33-day cessation, while the expression of *Mmp9* was seemingly unaltered by CS or cessation status. *Gpx1* expression was significantly reduced following CS, which persisted following a 33-day cessation, whereas, the expression of *Nox2* was increased following 3 and 10 day cessation but returned to sham level at day 33. Cessation significantly suppressed CS-induced *Nox1* expression, whilst, the CS-induced suppression of *Nox4* was unaltered by cessation.

### Hippocampal-dependent working memory persisted despite 33 days CS cessation.

**Fig. 2:**
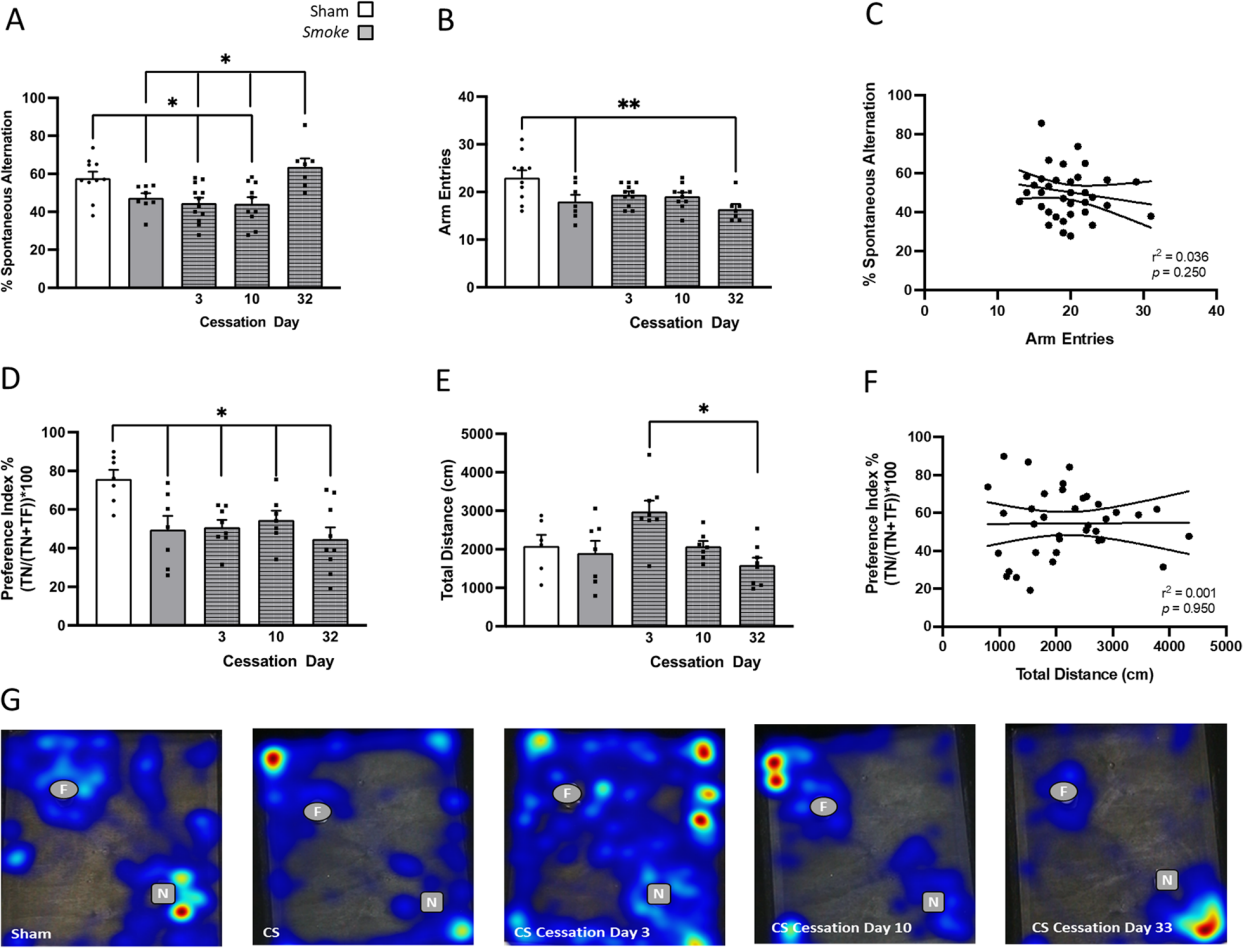
Cigarette smoke (CS) cessation reverses hippocampal-dependent spatial but not working memory impairments. **A** Spontaneous alternation percentage in Y-maze (*F*_(4,42)_ = 6.340, *p* = 0.0004; *n* = 7–11); **B** Y-maze arm entries (*F*_(4,40)_ = 4.482, *p* = 0.0004); **C** correlation between arm entries and spontaneous alternation in Y-maze (*r*_(43)_ = 0.0306, *p* = 0.250); **D** novel objection recognition (NOR) task preference index (*F*_(4,33)_ = 4.829, *p* = 0.0035; *n* = 7–9); **E** total distance (cm) travelled in NOR (*F*_(4,31)_ = 3.201, *p* = 0.0260; *n* = 7–9); **F** correlation between preference index and total distance (cm) in mice exposed to room air (sham), CS or CS cessation for either 3 days, 10 days or 33 days (*r*_(34)_ = 0.0001, *p* = 0.953); **G** heatmaps illustrating familiar object (circle) and novel object (square) exploration in the NOR. Heatmaps were generated in Ethovision using the over-heatmap setting allowing comparison between the representative animals. Data are expressed as mean + SEM. *p < 0.05, **p < 0.01

Smoke exposure induced spatial memory impairments, with a reduction in the percentage sY-maze task which remained evident up until 10 days but was resolved by 33 days post-cessation (Fig. 2A). CS-exposed mice had a noticeable decrease in arm entries compared to the sham counterparts (Fig. 2B), however, there was no correlation between spontaneous alternation and the number of arm entries made (Fig. 2C). Moreover, sham mice spent more time exploring the novel object (high preference index) indicating appropriate recall of the NOR task, whereas, CS-exposed mice were unable to differentiate between the novel and familiar object (neutral preference index; Fig. 2D, G) suggesting working memory impairment and this was not resolved following a 33-day cessation. Acute CS cessation for 3 days increased total distance travelled compared to 33 days cessation, however, the overall lack of difference in total distance travelled during the NOR task (Fig. 2E, F) is indicative that the working memory impairment in CS-exposed mice were not associated with an altered locomotor activity.

### Prolonged CS cessation induces microglial activation

**Fig. 3:**
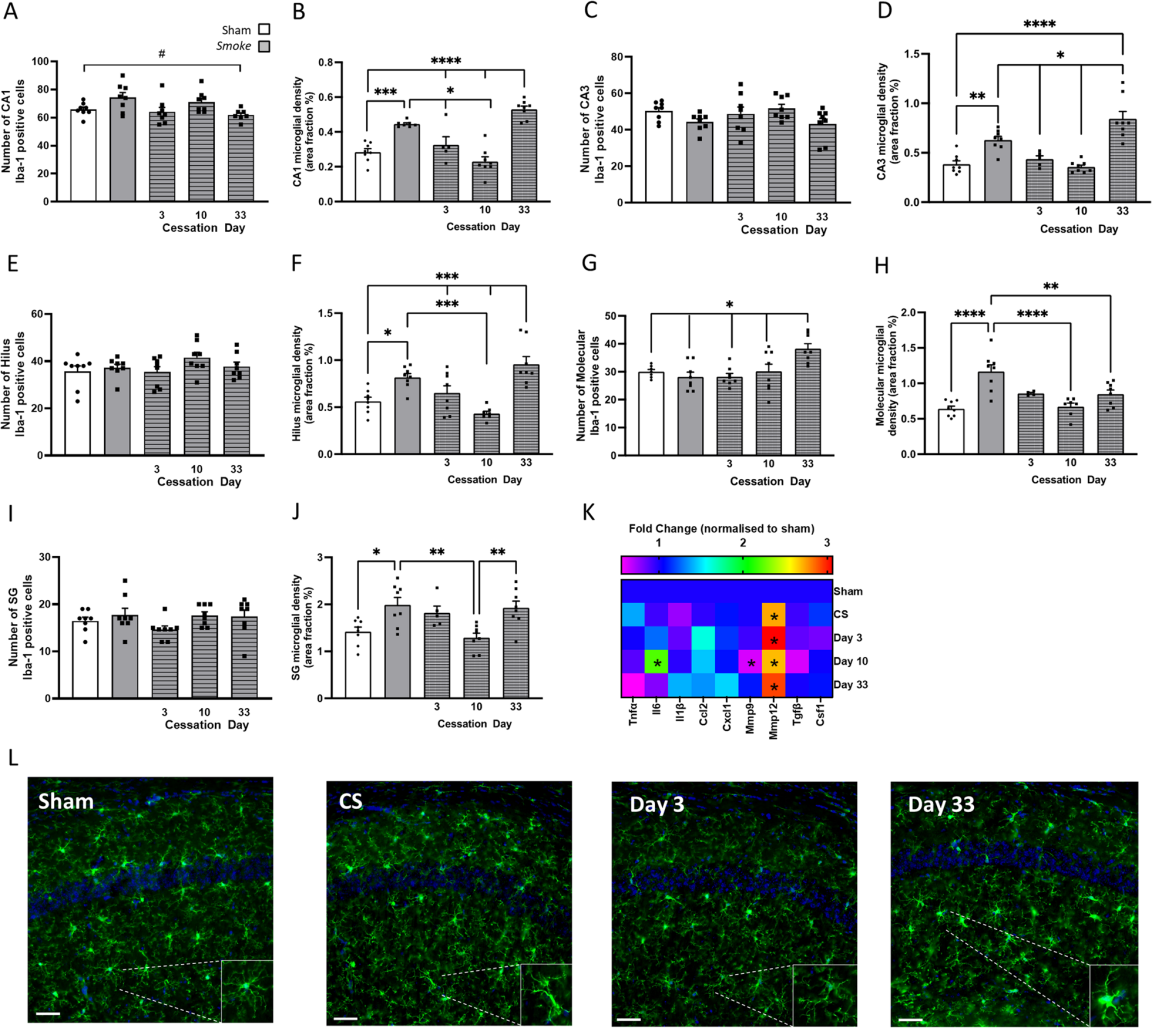
Cigarette smoke (CS) cessation alters microglial morphology. Microglial area per cell were increased throughout the hippocampus by CS exposure and microglia were further exacerbated 33 days after smoke cessation. **A–J** Ionised calcium binding adaptor molecular (Iba-1) labelling through the CA1, CA3, dentate gyrus (DG) hilus, DG molecular and DG subgranular/granular regions of the hippocampus. **A**, **B** CA1 (number: *F*_(4,34)_ = 3.652, *p* = 0.0140; area per cell: *F*_(4,31)_ = 28.55, *p* < 0.0001); **C**, **D** CA3 (area per cell: *F*_(4,32)_ = 20.84, *p* < 0.0001); **E**, **F** dentate gyrus hilus (Area per cell: *F*_(4,34)_ = 11.96, *p* < 0.0001); **G**, **H** molecular (number: *F*_(4,34)_ = 5.725, *p* = 0.0012; area per cell: *F*_(4,30)_ = 11.63, *p* < 0.0001); **I**, **J** subgranular/granular (area per cell: *F*_(4,32)_ = 6.222, *p* = 0.0008). **K** Gene expression of cytokines and chemokines in the hippocampus (Interleukin-6 [*Il6*]: *F*_(4,28)_ = 3.086, *p* = 0.0318; *Matrix Metalloproteinase [Mmp]9*: *F*_(4,27)_ = 6.152, *p* = 0.0012; *Mmp12*: *F*_(4,28)_ = 2.855, *p* = 0.042). **L** Representative photomicrographs of the CA1 from room air (sham) and CS-exposed mice illustrating differences in area per cell of Iba-1-stained cells. Scale bars = 50 µm. Data are expressed as mean + SEM (*n* = 5—8 per group). # *p* < 0.05 effect of exposure; * *p* < 0.05; ** *p* < 0.01; *** *p* < 0.001; **** *p* < 0.0001

CS exposure caused an increase in microglial numbers in the CA1 region of the hippocampus when compared to sham mice (Fig. 3A); however, did not differ in other regions (Fig. 3C, E, G, I). Cessation of 33 days increased Iba-1-positive staining in the molecular region of the dentate gyrus suggesting an increased number of microglia (Fig. 3G). CS exposure led to microglial activation as seen by an increased microglial cell area in all regions of the hippocampus (Fig. 3). Cessation initially suppressed microglial activation (at day 3 and 10), but eventually resulted in an increased microglial activation (Fig B, D, F, J). Like that of the lung profile, there was a significant increase in *Mmp12* expression following CS exposure which remained elevated despite CS cessation (Fig. 3K).

### Cessation was unable to resolve established hippocampal oxidative stress or synaptophysin dysfunction by CS exposure

**Fig. 4:**
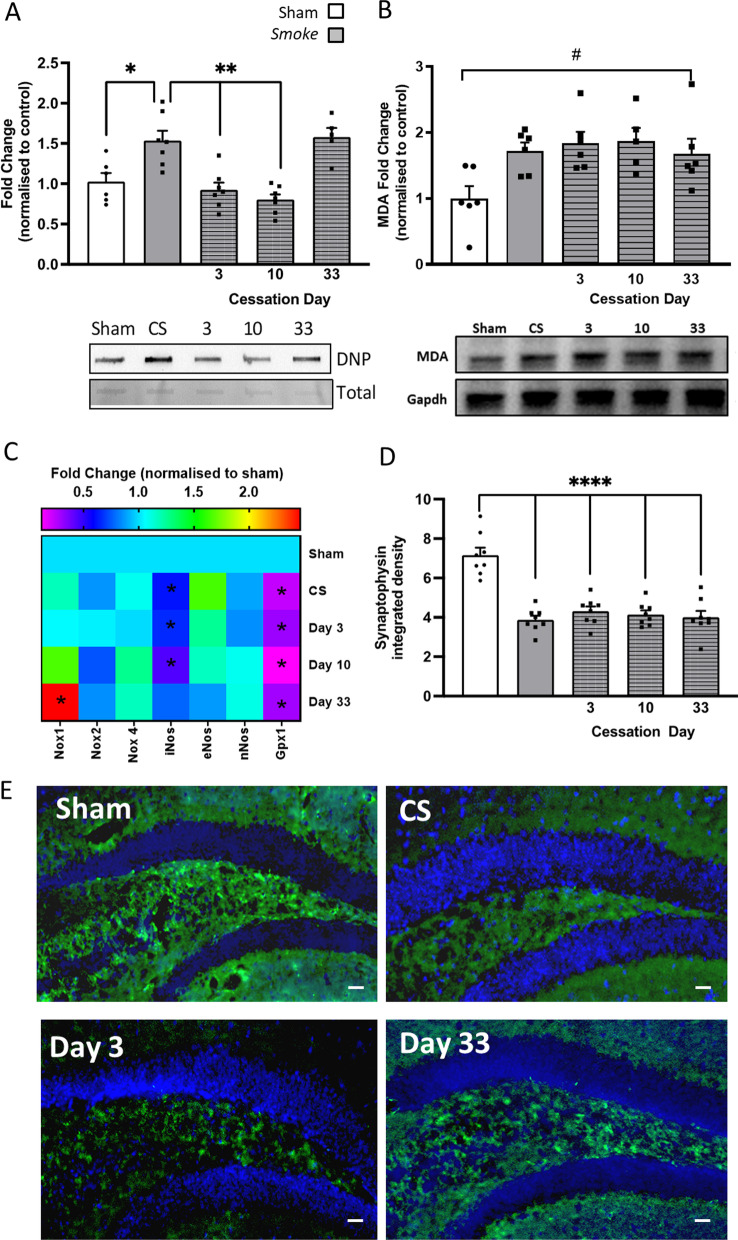
Cigarette smoke (CS) cessation increases hippocampal oxidative stress. **A** Protein carbonylation (*F*_(4,27)_ = 12.55, *p* < 0.0001; n = 6-7 per group); **B** lipid peroxidation (*F*_(4,24)_ = 3.721, *p* = 0.0171; n = 5-6 per group); **C** gene expression of oxidative markers in the hippocampus (NADPH oxidase [*Nox*]*1*: *F*_(4,24)_ = 4.263, *p* = 0.0084; *Nox4*: *F*_(4,28)_ = 9.330, *p* < 0.0001; Inducible nitric oxide synthase [*iNos*]: *F*_(4,29)_ = 4.474, *p* = 0.0061; Glutathione peroxidase [*Gpx*]1: F_(4,25)_ = 20.82, *p* < 0.0001; n = 6 per group). **D** Synaptophysin labelling in the dentate gyrus (DG) hilus region of the hippocampus (*F*_(4,35)_ = 23.88, *p* < 0.0001; *n* = 7–8 per group). **E** Representative photomicrographs of the DG hilus from room air (sham) and CS-exposed mice illustrating differences in synaptophysin density. Data are expressed as mean + SEM. # *p* < 0.05 main effect of exposure; * *p* = 0.05; ** *p* < 0.01, **** *p* < 0.0001

Exposure to CS caused an increase in protein carbonylation of hippocampal tissue compared to sham mice (Fig. 4A). Similar to the microglial profile, the CS-induced protein carbonylation was initially lower compared to CS mice but re-emerged following 33 days of cessation. CS exposure caused an increase in MDA expression which was unaltered by cessation (Fig. 4B). *Nox1* expression was significantly elevated following 33 days of cessation when compared to sham-exposed mice. Inducible nitric oxide synthase (*iNos*) expression was reduced by CS exposure and eventually normalised to sham level following 33 days of cessation. Like the lung profile, *Gpx1* in CS-exposed and CS cessation mice was suppressed when compared to the shams. The presynaptic protein, synaptophysin, was reduced in the hippocampus of CS-exposed and cessation mice compared to sham mice (Fig. 4D, E).

### Ebselen partially resolves CS-induced pulmonary inflammation.

**Fig. 5:**
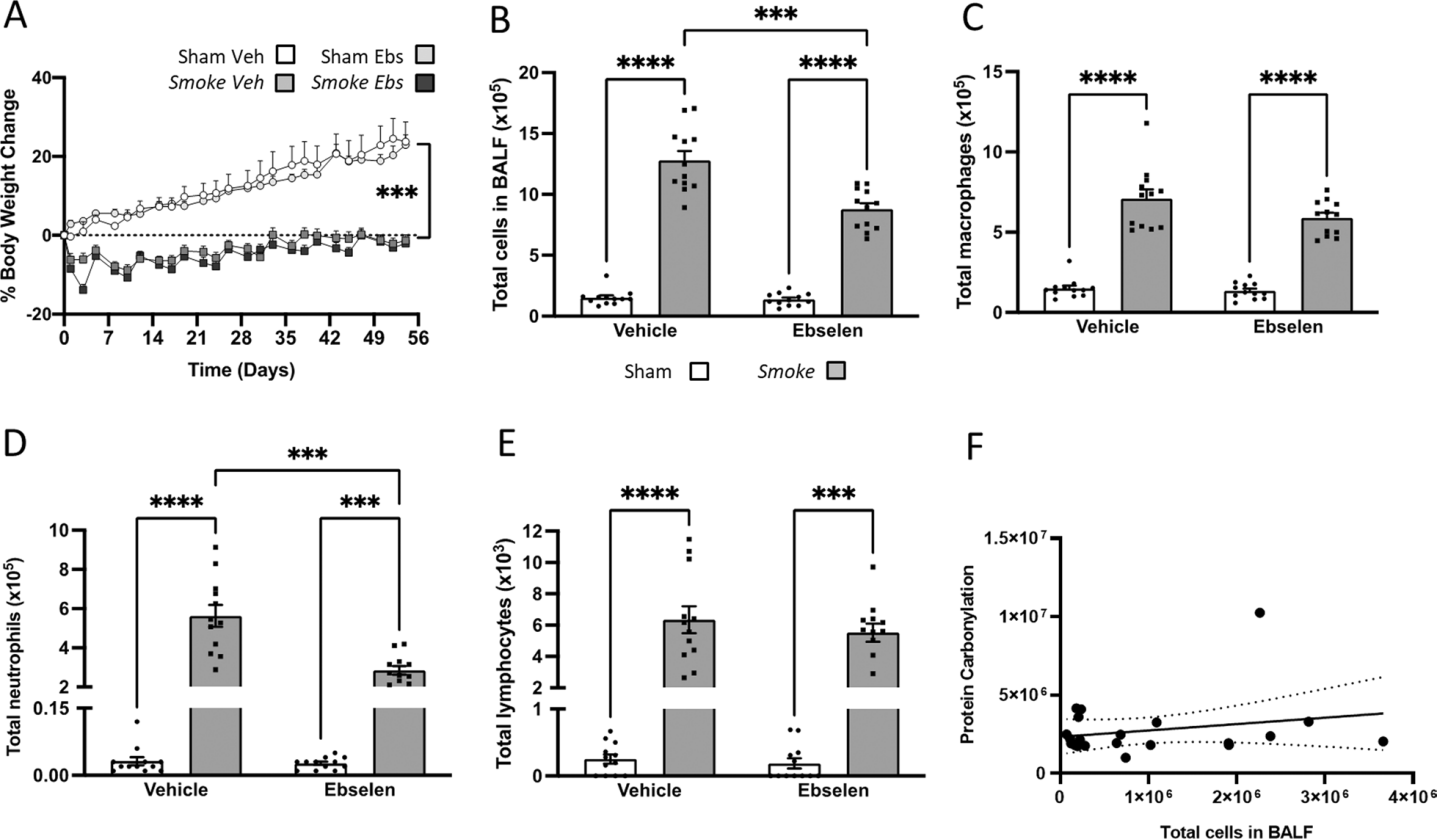
Cigarette smoke (CS)-induced bronchoalveolar lavage fluid (BALF) cellularity was partially reversed by ebselen treatment. Mice were exposed to CS or room air (sham) concomitant with ebselen treatment for 8 weeks. **A** Body weight percentage change (*n* = 12 per group). **B** Total number of cells (interaction between exposure and treatment: F_(1,11)_ = 20.88, *p* = 0.0008) (**C**); macrophages (main effect of exposure: *F*_(1,11)_ = 246.5, *p* < 0.0001) (**D**); neutrophils (interaction between exposure and treatment: *F*_(1,11)_ = 19.57, *p* = 0.0001) (**E**); and lymphocytes (main effect of exposure: *F*_(1,11)_ = 173.9, *p* < 0.0001) in BALF (*n* = 12 per group). **F** BALF protein carbonylation (*r*_(20)_ = 0.0523, *p* = 0.306; *n* = 12 per group). Data are expressed as mean + SEM. *** *p* < 0.001, **** *p* < 0.0001

To determine how CS exposure elicits pulmonary inflammation and elevated oxidative stress levels damaging the brain, we pharmacologically targeted oxidative stress using a GPx mimetic, ebselen, to enhance the removal of ROS. Ebselen retained body weight to that of CS vehicle mice (Fig. 5A) and also markedly attenuated the CS-induced lung inflammation evidenced by the reductions in neutrophil recruitment (Fig. 5B–E). To ascertain whether the attenuated inflammation of the lungs was associated with a dampened oxidative stress from ebselen administration, we assessed protein oxidation of the BALF. Our analysis revealed that there was no correlation between protein oxidation and the total number of immune cell infiltration suggesting that both 5% CM cellulose and ebselen was able to antagonise the oxidative burden evoked by CS (Fig. 5F).

### Ebselen reverses hippocampal-dependent memory impairment following CS exposure

**Fig. 6:**
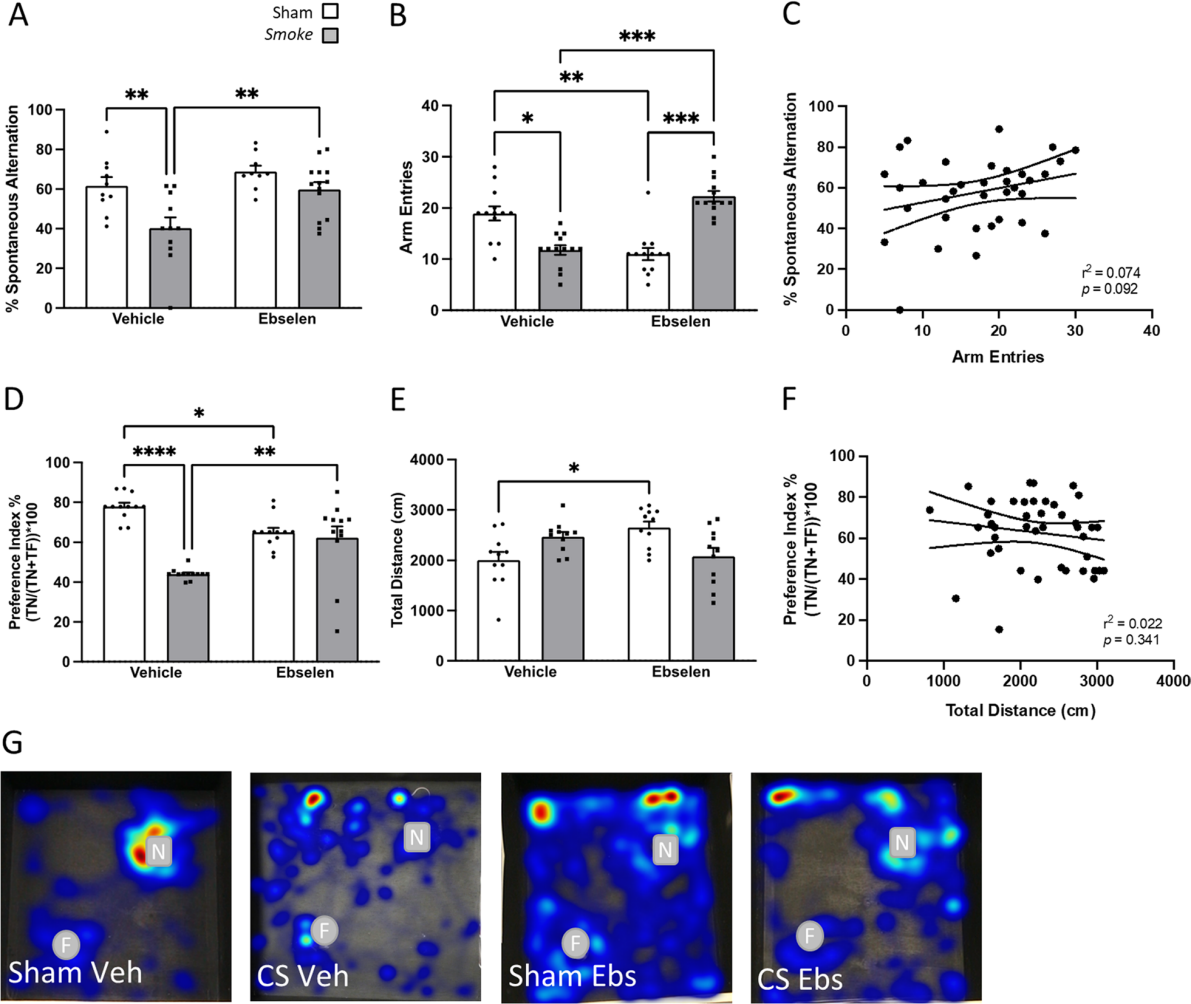
Prophylactic ebselen treatment reverses hippocampal-dependent memory impairments. **A** Spontaneous alternation percentage in Y-maze (significant effect of exposure: *F*_(1,23)_ = 22.60, *p* < 0.0001; significant effect of treatment: *F*_(1,23)_ = 9.792, *p* = 0.0047; *n* = 7–11); **B** Y-maze arm entries (significant interaction between exposure and treatment: *F*_(1,12)_ = 44.56, *p* < 0.0001); **C** correlation between arm entries and spontaneous alternation in Y-maze (*r*_(37)_ = 0.074, *p* = 0.092); **D** novel objection recognition (NOR) task preference index (*F*_(1,11)_ = 29.48, *p* = 0.0002 *n* = 12); **E** total distance (cm) travelled in NOR (*F*_(1,10)_ = 12.26, *p* = 0.0057; *n* = 11); **F** correlation between preference index and total distance (cm) in mice exposed to room air (sham), smoke and/or vehicle or ebselen (*r*_(41)_ = 0.022, *p* = 0.341); **G** heatmaps illustrating familiar object (circle) and novel object (square) exploration in the NOR. Heatmaps were generated in Ethovision using the over-heatmap setting allowing comparison between the representative animals. Data are expressed as mean + SEM. *p < 0.05; **p < 0.01; ***p < 0.001; ****p < 0.0001

CS exposure reduced the percentage spontaneous alternation suggesting spatial memory impairment, which was completely reversed by ebselen treatment (Fig. 6A). CS-exposed mice were also found to have reduced number of arm entries (Fig. 6B), but this was unlikely to be a result of reduced locomotor activity, reflected by the lack of correlation between the two (Fig. 6C). Moreover, concurrent ebselen treatment with CS exposure was able to completely block the onset of working memory deficits (Fig. 6D, G), which was also independent of locomotor activity, suggesting inhibition of oxidative stress protects against hippocampal-dependent memory loss by CS exposure (Fig. 6E, F). We also see that a slightly increased total distance travelled in the NOR task (Fig. 6E), but this was not correlated with working (Fig. 6F) memory.

### Microglial profiles are unchanged in CS-exposed mice irrespective of ebselen treatment

**Fig. 7:**
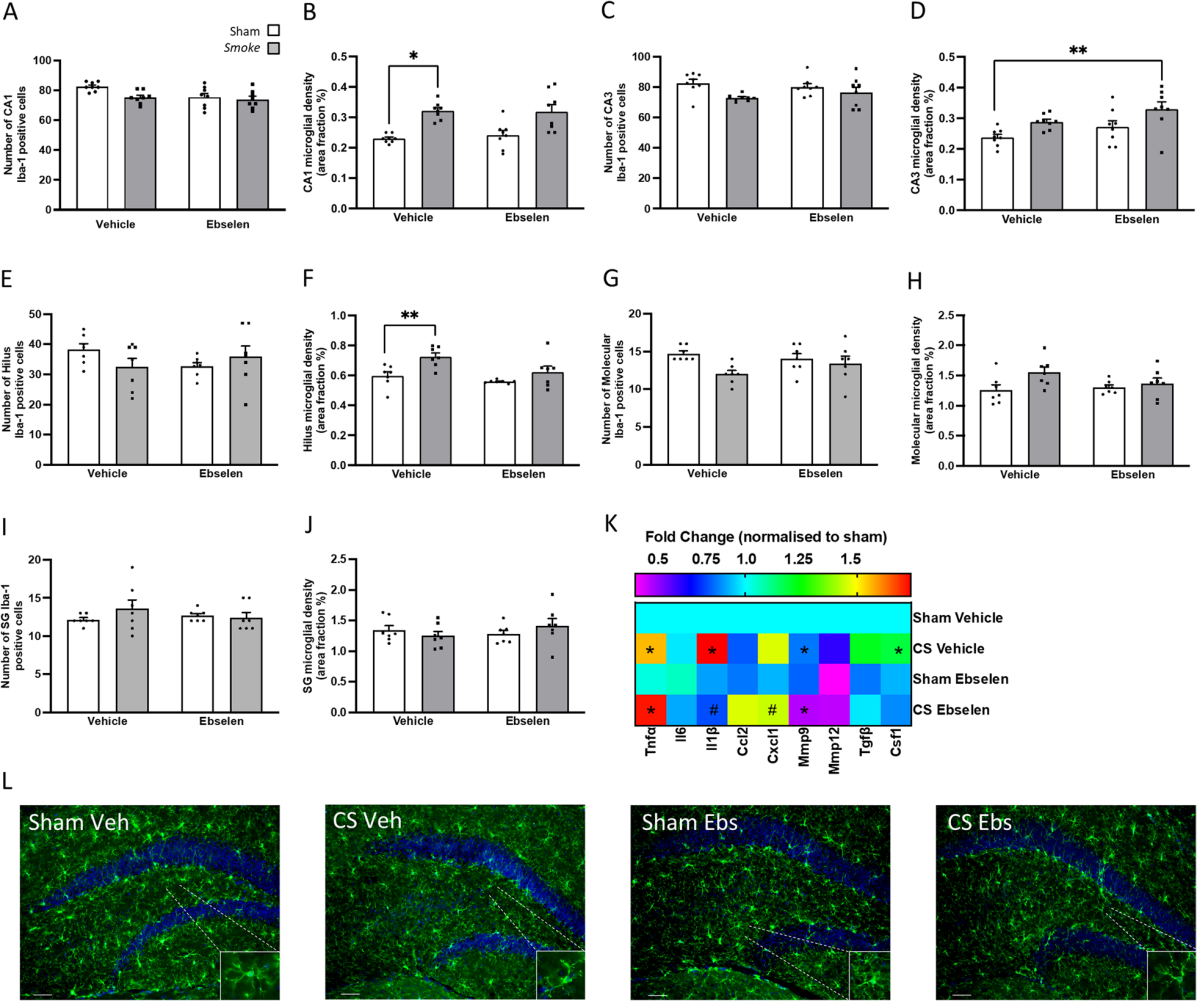
Prophylactic ebselen treatment does not reverse hippocampal microglial profile. **A**–**J** Ionised calcium binding adaptor molecular (Iba-1) labelling through the CA1, CA3, dentate gyrus (DG) hilus, molecular and subgranular/granular regions of the hippocampus. **A**, **B** CA1 (area per cell: main effect of exposure: *F*_(1,7)_ = 45.45, *p* = 0.0002; n = 5–8 per group); **C**, **D** CA3 (area per cell: main effect of exposure: *F*_(1,7)_ = 45.06, *p* = 0.0003 and main effect of treatment: *F*_(1,7)_ = 6.164, *p* = 0.042;); **E**, **F** DG hilus (area per cell: main effect of exposure: *F*_(1,6)_ = 6.902, *p* = 0.039 and main effect of treatment: *F*_(1,7)_ = 24.15, *p* = 0.002); **G**, **H** molecular; **I**, **J** subgranular. **K** Gene expression of cytokines and chemokines in the hippocampus (Tumor necrosis factor [*Tnf*]:  main effect of exposure: *F*_(1,5)_ = 128.8, *p* < 0.0001; Interleukin [*Il*]*1b*: main effect of exposure: *F*_(1,5)_ = 29.94, *p* = 0.0028 and main effect of treatment: *F*_(1,5)_ = 10.05, *p* = 0.0248; C-X-C motif chemokine ligand [*Cxcl*]1: main effect of exposure: *F*_(1,5)_ = 12.22, *p* = 0.0174; Matrix Metalloproteinase [*Mmp*]9: main effect of exposure: *F*_(1,5)_ = 40.22, *p* = 0.0014 and main effect of treatment: *F*_(1,5)_ = 9.852, *p* = 0.0257; Colony-Stimulating Factor [*Csf*]: main effect of exposure: *F*_(1,5)_ = 7.478, *p* = 0.0141; *n* = 6 per group). **L** Representative photomicrographs of the CA1 from room air (sham) and cigarette smoke-exposed mice illustrating differences in area per cell of Iba-1-stained cells. Scale bars = 50 µm. Data are expressed as mean + SEM. * *p* < 0.05

CS exposure evoked no significant change in microglial number in the hippocampus (Fig. 7A, C, E, G, I) similar to that of the cessation cohort (Fig. 3). CS exposure increased microglia area per cell in the CA1 (Fig. 7B). In the CA3 and hilus region of the dentate gyrus, CS exposure increased area per cell compared to the sham mice and this was further augmented by the ebselen treatment (Fig. 7D, F). There were no differences in microglia area per cell in the molecular and sub-granular regions of the dentate gyrus irrespective of exposure or treatment (Fig. 7H,  J). Irrespective of ebselen treatment, CS exposure significantly increased *Tnfα*, *Cxcl1*, *Il1β* and *Csf1* expressions compared to sham mice, but *Il1β* and *Mmp9* expression were reduced following ebselen treatment (Fig. 7K).

### Ebselen treatment preserved synaptophysin expression in the hippocampus

**Fig. 8:**
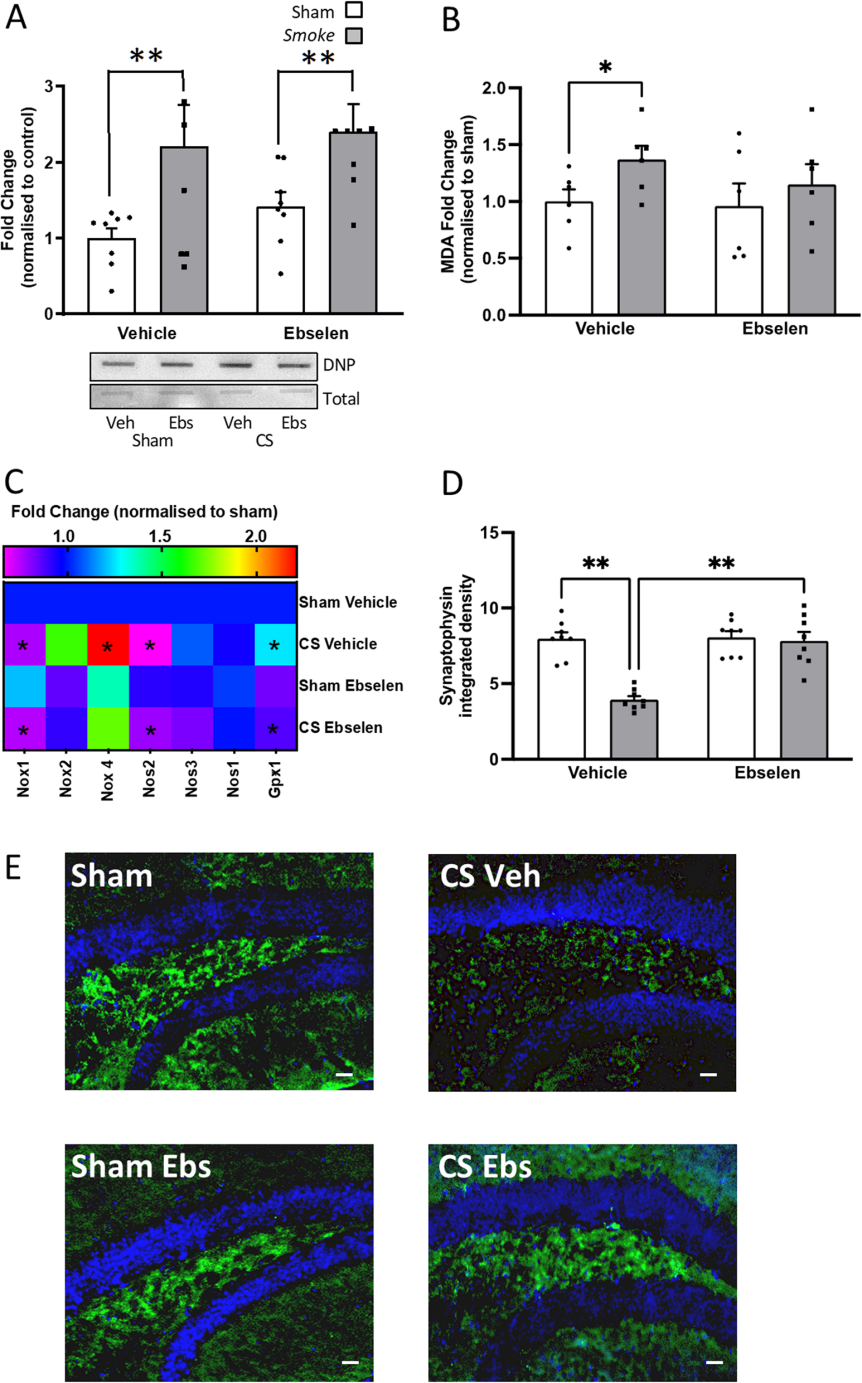
Ebselen prevents cigarette smoke (CS)-induced synaptophysin deficits. **A** Protein carbonylation (main effect of exposure: *F*_(1,28)_ = 10.26, *p* = 0.0034; *n* = 6 per group); **B** lipid peroxidation (main effect of exposure: *F*_(1,5)_ = 12.37, *p* = 0.018; *n* = 6 per group); **C** gene expression of oxidative markers in the hippocampus (NADPH oxidase [*Nox1*]: main effect of exposure: *F*_(1,5)_ = 350.46, *p* = 0.0019; *Nox4*: main effect of exposure: *F*_(1,5)_ = 6.819, *p* = 0.0476; Nitric oxide synthase [*Nos2 / iNos*]: main effect of exposure: *F*_(1,5)_ = 9.311, *p* = 0.0028; Glutathione peroxidase [*Gpx*]1: main effect of treatment: *F*_(1,5)_ = 29.72, *p* = 0.0028; *n* = 6 per group). **D** Synaptophysin labelling in the dentate gyrus hilus region of the hippocampus (*F*_(1,7)_ = 17.42, *p* = 0.0042; *n* = 7–8 per group). Data are expressed as mean + SEM. * *p* < 0.05; ** *p* < 0.01

CS exposure alone caused an increase in total protein carbonylation when compared to sham-treated mice (Fig. 8A) and ebselen treatment did not resolve this. MDA levels were also enhanced upon CS exposure which returned to sham levels following ebselen treatment (Fig. 8B). CS-exposure significantly suppressed *Nox1* and *Nos2* expression when compared to sham mice, which was unaltered by ebselen treatment. Ebselen was able to restore the CS-induced elevations in *Nox4* and *Gpx1* expression to that of sham mice (Fig. 8C). Whilst the hippocampal protein carbonylation profile remained elevated, we found that ebselen completely restored the CS-induced reduction in synaptophysin density (Fig. 8D, E).

## Discussion

The present study suggested that CS exposure caused lung inflammation, hippocampal neuroinflammation, suppression of synaptophysin expression, and spatial and working memory deficits. 33 days of cessation resolved the apparent pulmonary inflammation, however, expression of pro-inflammatory mediators remained elevated in lung tissues, which is consistent with previously published literature [38]. These cognitive deficits and reductions in a key mediator of synaptogenesis. synaptophysin persisted well after cessation, suggesting these alterations to the CNS are not transient and may be responsible for the long-term risk of neurodegenerative diseases. For this reason, we specifically targeted the pulmonary oxidative profile via concomitant ebselen treatment to delineate the role of oxidative stress in cognitive decline. Ebselen completely prevented CS-induced neurocognitive dysfunction and our molecular analysis found that this is attributed to a restoration of synaptophysin.

Smoking cessation studies focusing on cognitive integrity in people with COPD are limited, with many studies excluding participants with COPD from cohort analysis, which could be due to the numerous comorbidities considered as an exclusion criterion [39, 40]. Research has shown that CS cessation in non-COPD participants may increase the risk of accelerated cognitive decline and dementia [23, 39–41]. Two recent studies found that compared with current non-COPD smokers, long-term quitters (> 9 years) had a decreased risk of dementia, however, this is not the case for short-term quitters (< 9 years) [39, 42]. Conversely, others revealed that smokers who had quit for longer than 3 years had reduced risk of dementia comparable with that of never smokers [41]. Others have shown that long-term smoking cessation of greater than 20 years is necessary to fully ameliorate the risk of cognitive impairment and dementia [23, 43]; however, the mechanisms underlying these outcomes remain unclear. We have shown that 8 weeks of CS exposure in BALB/c mice recapitulates key aspects of lung pathologies to that observed in human COPD along with the persistent memory deficits, allowing us to investigate the potential underlying mechanism.

Nicotine is the major psychoactive component of cigarettes involved in the neurobiological effects underlying the sustainment, and reinforcement of smoking. It has become clear that smoking cessation can lead to nicotine withdrawal symptoms including alterations in attention, working and episodic memory, cognitive flexibility and contextual learning processes [44–49]. It is advantageous to consider that previous nicotine studies utilised chronic, continuous administration of nicotine via subcutaneous-implanted osmotic pumps, which is distinctly different from the human smoking pulmonary kinetic pattern and blood concentration of nicotine in smokers [50]. Our study shows a decline in working memory following 8 weeks of CS exposure, which persisted beyond 33 days post-cessation suggesting these neurocognitive impairments may be closely related to pulmonary inflammation elicited by CS rather than nicotine withdrawal symptoms, which have been shown to be fully resolved by 8-day post-nicotine withdrawal. Alongside this, studies have found that CS and e-cigarette cessation for more than 30 days markedly impaired spatial and visual memory [51, 52]. Similarly, mice exposed to nicotine-free e-cigarettes have impaired working memory performance, further emphasising that cognitive impairments may still occur without nicotine [53].

In our model, chronic smoke exposure promoted sustained hippocampal microglial activation that is unaltered by cessation. Consistent with our notion that CS-induced COPD alters the microglial morphology to an ameboid state, an acute treatment of the tobacco-specific procarcinogen compound, 4-*N*-methyl-*N*-nitrosamino-1-(3-pyridyl)-1-butanone in mice, for as little as 4 days, can induce robust changes in hippocampal microglial morphology [54]. This reinforces the concept that pulmonary inflammation is likely to be responsible for the activation and modulation of microglial cells. Clinical studies have opposingly shown that smokers have less microglial activation than non-smokers using [11C]DAA1106; a radiotracer for translocator protein (TSPO), an indicator of microglial activation [55–58]. Interestingly, TSPO deficiency suppresses mitochondrial oxidative phosphorylation and glycolysis in mice, resulting in overall mitochondrial dysfunction [59]. Indeed, CS exposure is known to induce a maladaptive mitochondrial hyperfusion response in alveolar epithelial cells [60] and beyond the pulmonary system [61]. Thus, one explanation for the clinical findings is that [11C]DAA1106 is highly metabolised in smokers compared to non-smokers, rather than there being a suppression of microglial activation. Going forward, it will be imperative to evaluate whether pharmacologically targeting microglia both during CS exposure and cessation periods could improve cognitive outcomes. For instance, pharmacological inhibition of microglial activation using minocycline, has been shown to improve cognitive function [62, 63]. Moreover, stable COPD patients given a tetracycline analogue for 4 weeks showed an improvement in lung function and a reduction in systemic inflammation compared to vehicle-treated COPD patients, thus, it may be capable of attenuating neuroinflammation [64].

Oxidative stress is a key driver of CS-induced lung damage and has recently been proposed as a fundamental driver of neurodegeneration and cognitive dysfunction [65], however, there is limited literature on the impact of CS-induced oxidative stress in the brain. We show that cognitive impairment is associated with elevated levels of protein carbonylation and lipid peroxidation as well as down-regulated *Gpx1* expression in the hippocampus of CS exposed and CS cessation mice. Studies have shown increased gene expression of pro-oxidants such as *iNos*, *Nox4*, and *Nox2* in the whole brain following CS exposure, which are typically triggered as a secondary response to oxidative stress [66]. Moreover, CS exposure induces a reduction of cytoplasmic staining of transcription factor Nrf2 (nuclear factor [erythroid-derived 2]-like 2) in rats [66] and mice [67] which is a master regulator of a myriad of cellular antioxidants, thus suggesting the dual action of CS exposure on enhancing oxidant generation and suppression of cellular defence.

Given that excessive oxidative stress is often linked to microglial activation [68] and that microglial cells are a major cellular source of ROS under inflammatory states [69, 70], it is tempting to speculate that a prophylactic antioxidant treatment may attenuate the oxidative burden and improve CS-induced cognitive dysfunction. A prophylactic treatment with selenium and Vitamin E concomitant with CS exposure for 20 weeks in mice was sufficient to prevent impairments in whole brain’s antioxidant defence system [71]. It must be noted that previous studies have assessed whole brain tissue, rather than individual regions. We found that hippocampal lipid peroxidation is significantly reduced following antioxidant treatment, however, protein carbonylation is not. The hippocampus is highly susceptible to oxidative insults [72], thus, it seems highly plausible that ebselen may be able to reduce the CS-induced oxidative burden globally, however, hippocampal oxidative stress may remain elevated alongside the elevated microglial profile. This is not unprecedented given that the attenuation of pulmonary inflammation following ebselen administration is largely due to a reduction in neutrophilic infiltration [7, 10], leading us to postulate that ebselen’s memory enhancing ability may be exerted via a microglial-independent mechanism which targets synaptic integrity in smokers. Synapses are formed by the functional contact of presynaptic axonal terminals with postsynaptic dendritic processes allowing for the efficient transmission of signal [73, 74]. It is apparent that aberrant synaptic plasticity, due to abnormal expression of synaptophysin and the postsynaptic scaffolding protein, PSD-95, in the hippocampus, is functionally related to cognitive decline and Alzheimer’s disease [75–77]. The loss of pre/post-synaptic proteins, progressive loss of synaptic density and the subsequent cognitive decline is typified by an increase in reactive astrocytic expression, alongside microglial activation [78, 79]. We, and others, have demonstrated that CS exposure induces a loss of synaptic integrity, via a reduction in synaptophysin in the hilus region of the dentate gyrus [80] and importantly this is not reversible even after 33 days CS cessation. Moreover, concomitant ebselen treatment prevented the loss of synaptic integrity by retaining synaptophysin density in CS-exposed mice to that of sham mice.

## Conclusions

CS cessation remains one of the most effective strategies for preventing and reducing the progressive decline in lung function attributable to COPD [18–21, 81, 82]. Clinical research indicates that long-term non-COPD quitters have improved cognitive integrity compared to current smokers [22–24, 41]. We are the first to show that neuroinflammation persists in spite of cessation and this is associated with continuous cognitive impairments, even when pulmonary inflammation has subsided. Moreover, we demonstrate that a prophylactic pharmacological treatment with ebselen can preserve cognitive function and prevent the loss of synaptophysin, despite the sustained microglial activation following CS exposure. Mitigating the pulmonary inflammation and the potential “spill-over” into the CNS could halt the neuroinflammatory profile and the associated cognitive decline in CS-induced COPD. Therefore, it is clear from our study that CS cessation alone is not enough to improve neuroinflammation and cognition and that future research should focus on investigating CS cessation with concomitant pharmacological interventions, particularly antioxidants, which we have shown to target both pulmonary and systemic inflammation and the associated neurological comorbidities.

## Data Availability

The datasets supporting the conclusions of this article are available upon request.

## Abbreviations

BALF: Bronchoalveolar lavage fluid
Ccl: C–C motif chemokine ligand
COPD: Chronic obstructive pulmonary disease
CS: Cigarette smoke
Cxcl: C–X–C motif chemokine ligand
Gpx: Glutathione peroxidase
IL: Interleukin
iNOS: Inducible nitric oxide synthase
MDA: Malondialdehyde
Mmp: Matrix metalloproteinase
NOR: Novel object recognition
NOX: NADPH oxidase
qRT-PCR: Quantitative real-time-PCR
ROS: Reactive oxygen species
sY-maze: Spontaneous alternation in the Y-maze
Tnf: Tumour necrosis factor
TSPO: Translocator protein
